# Early Diagnosis of Type 2 Diabetes Based on Near-Infrared Spectroscopy Combined With Machine Learning and Aquaphotomics

**DOI:** 10.3389/fchem.2020.580489

**Published:** 2020-12-07

**Authors:** Yuanpeng Li, Liu Guo, Li Li, Chuanmei Yang, Peiwen Guang, Furong Huang, Zhenqiang Chen, Lihu Wang, Junhui Hu

**Affiliations:** ^1^College of Physical Science and Technology, Guangxi Normal University, Guilin, China; ^2^Guangxi Key Laboratory Nuclear Physics and Technology, Guangxi Normal University, Guilin, China; ^3^Guangdong Hongke Agricultural Machinery Research & Development Co., Ltd., Guangzhou, China; ^4^First Affiliated Hospital of Jinan University, Guangzhou, China; ^5^Guangdong Provincial Key Laboratory of Optical Sensing and Communications, Department of Optoelectronic Engineering, Jinan University, Guangzhou, China

**Keywords:** type 2 diabetes, early diagnosis, near-infrared spectroscopy(NIR), support vector machine (SVM), aquaphotomics

## Abstract

Early diagnosis is important to reduce the incidence and mortality rate of diabetes. The feasibility of early diagnosis of diabetes was studied via near-infrared spectra (NIRS) combined with a support vector machine (SVM) and aquaphotomics. Firstly, the NIRS of entire blood samples from the population of healthy, pre-diabetic, and diabetic patients were obtained. The spectral data of the entire spectra in the visible and near-infrared region (400–2,500 nm) were used as the research object of the qualitative analysis. Secondly, several preprocessing steps including multiple scattering correction, variable standardization, and first derivative and second derivative steps were performed and the best pretreatment method was selected. Finally, for the early diagnosis of diabetes, models were established using SVM. The first overtone of water (1,300–1,600 nm) was used as the research object for an aquaphotomics model, and the aquagram of the healthy group, pre-diabetes, and diabetes groups were drawn using 12 water absorption patterns for the early diagnosis of diabetes. The results of SVM showed that the highest accuracy was 97.22% and the specificity and sensitivity were 95.65 and 100%, respectively when the pretreatment method of the first derivative was used, and the best model parameters were c = 18.76 and g = 0.008583.The results of the aquaphotomics model showed clear differences in the 1,400–1,500 nm region, and the number of hydrogen bonds in water species (1,408, 1,416, 1,462, and 1,522 nm) was evidently correlated with the occurrence and development of diabetes. The number of hydrogen bonds was the smallest in the healthy group and the largest in the diabetes group. The suggested reason is that the water matrix of blood changes with the worsening of blood glucose metabolic dysfunction. The number of hydrogen bonds could be used as biomarkers for the early diagnosis of diabetes. The result show that it is effective and feasible to establish an accurate and rapid early diagnosis model of diabetes via NIRS combined with SVM and aquaphotomics.

## Introduction

Pre-diabetes refers to abnormal fasting glucose or impaired glucose tolerance that has not yet reached the diagnostic criteria for diabetes. It is the only reversible stage in the course of type 2 diabetes (Yu et al., 2013). One study has indicated that there will be 472 million people in the world with diabetes by the year 2025 (Xiaomin et al., 2016). Patients have no specific symptoms in the early stages of type 2 diabetes. Once diagnosed, the majority of cases have serious complications associated with them that will affect the patients' physical and mental health (Fukuda and Mizobe, 2017). Therefore, it is important to develop methods for the early diagnosis of type 2 diabetes so that appropriate diet and lifestyle interventions can be provided at an early stage to reduce the incidence of diabetes and control the condition of pre-diabetes.

At present, the screening test for pre-diabetes involves fasting plasma glucose (FPG), urine glucose, hemoglobin A1C (HbA1C), and gene testing. The detection of FPG and urine glucose is not easy to operate, is time-consuming, and has a low cost, but the missed diagnosis rate is high. The HbA1c detection method has little variability, and the result is not affected by eating time and short-term lifestyle factors. However, there is no unified diagnostic standard associated with the HbA1c detection method, which does not represent the current blood glucose level and easily results in misdiagnosis (Vajravelu and Lee, 2018). Genetic testing methods are varied and are selected according to particular requirements, but there are still legal and social ethics issues (Etchegary et al., 2010; Prior et al., 2012). Glucose tolerance test (OGTT) methods have the advantages of objectivity and accuracy, which is the “gold standard” for the diagnosis of pre-diabetes. However, the testing is complicated and time-consuming, causes great discomfort and increases the unnecessary psychological burden on patients, and is not suitable for large-scale population screening. Therefore, the development of fast, simple, and accurate methods is urgently required.

Patients with symptoms can undergo diagnostic methods, such as FPG, glucose tolerance tests, and glycated hemoglobin tests. However, these cannot be applied to large-scale screening (American Diabetes Association, 2012; Rosella et al., 2015; Mainous et al., 2016; Nakagami et al., 2017). Additionally, biomarkers have been investigated and applied to the early diagnosis of diabetes in a study by Lina et al. in 2013 that focused on the examination of whether there is a potential biomarker of T2DM in urine. The research showed that the expression of three types of polypeptides decreased in diabetes patients. It was further determined that these three polypeptides were fragments of histidine trimer nucleotide-binding protein 1 (HINT1), bifunctional aminoacyl tRNA synthetase (EPRS), and agrin precursor protein (CLU) and that they could be used as potential biomarkers for type 2 diabetes (Lina et al., 2013). In 2017, Hsiao-Feng et al. used laser doppler blood flow measurements and spectroscopic analysis to study different microcirculation effects and applied them to the early diagnosis of diabetes. Relative energy contribution and Doppler frequency shifts were found to decrease sequentially from the healthy group to the pre-diabetes group to the diabetes group. This shows that the relative energy contribution and Doppler frequency shift have a certain correlation with the progression of diabetes (Hsiao-Feng et al., 2017). In 2020, Yuanjie et al. used a wearable active acetone biosensor for the non-invasive diagnosis of pre-diabetes. Breath acetone on the order of ppm was measured, which showed that the sensor had a good response (Yuanjie et al., 2020). In new biomarker detection, laser doppler and acetone biosensors have been used in the early diagnosis of diabetes, and have made some progress, but these research results are very preliminary (Yuanjie et al., 2020).

Near-infrared spectroscopy (NIRS), as a non-destructive, rapid, and green analytical technique, has been widely used in the biomedical field (Workman, 1993; Beć et al., 2020). Metabolic or compositional changes occur during disease progression in most cases, beginning with abnormal changes in the molecular structure of tissue cells or humoral metabolism, and no obvious clinical symptoms are observed until the middle or late stages of disease onset. Consequently, analyzing the concentration and structural changes of proteins, fats, and water in human tissues, cells, and body fluids using NIRS, which is a more objective, reliable, and accurate tool has been proposed for the early diagnosis of diseases (Sakudo, 2016). The main component of blood is water, and other components include protein, lipid, sugar, and additional organic compounds. These substances have strong infrared activity, but the information of other components can be easily obscured owing to the strong absorption of water. Due to the influence of water absorption, NIRS has strong overlapping characteristics, and it is difficult to find the fingerprint features related to disease development. Besides, due to individual differences and instrument noise, it is difficult to detect subtle differences in the peak position of NIRS. To reduce the influence of these factors and extract useful information, it is necessary to combine spectral information with a machine learning method to establish a diagnostic model for the accurate diagnosis of pre-diabetes, which can reduce the influence of these factors and extract effective information (Huazhou et al., 2020).

In recent years, a novel approach called aquaphotomics has been proposed by Tsenkova (2005, 2009). This approach provides a new view of NIRS analysis. It allows analysis of NIR absorption changes of water and other substances as interference factors, and the changes in water absorption patterns associated with the occurrence and development of diseases can be determined by an extended water mirror approach (EWMA). Tsenkova et al. analyzed the number of body cells in the milk produced by cows with mastitis and healthy cows, and collected a large amount of milk component data and observed the changes in water absorption patterns in NIRS for the fast and accurate diagnosis of mastitis (Atanassova et al., 2009). Kinoshita et al. predicted whether a panda was in estrus by observing the changes in 12 water matrix coordinates—WAMACS in the urine of female pandas using the aquaphotomics method (Tsenkova, 2009; Kinoshita et al., 2012). For exploring the potential diagnostic information from serum samples, temperature-dependent near-infrared (NIR) spectroscopy was developed to obtain the spectral change of water reflecting the interactions in serum solution, and chemometric methods were employed to discriminate the patients of diabetes and heart disease. However, there have been no reports concerning the diagnosis of pre-diabetes using aquaphotomics.

The present work aims to develop a rapid and accurate diagnosis of pre-diabetes. A model for the diagnosis of pre-diabetes was established, which combined NIRS and a support vector machine (SVM). The changes in water absorption patterns in the blood of normal, pre-diabetic, and diabetic patients were extracted using the aquaphotomics method, which not only provides immediate insight for the occurrence and development of diabetes but also provides a novel method for the diagnosis of pre-diabetes that can hopefully be used for early diagnosis.

## Materials and Methods

### Materials and Sample Preparation

A total of 147 blood samples comprehensively diagnosed as healthy, pre-diabetic, or type 2 diabetic by 2-h post-load blood glucose (2hPG) of OGTT and FPG were collected from the Department of Endocrinology, First Affiliated Hospital of Jinan University. Peripheral blood samples were preserved (about 1 mL), kept in an anticoagulant tube (test tube treated with an anticoagulant to prevent blood from clotting), and stored in a −20°C refrigerator. All the peripheral blood samples are collected on the same day and spectral acquisition was performed immediately. Blood samples were collected from 53 healthy (24 males and 29 females, with an average age of 44 ± 12 years), 46 pre-diabetic (18 males and 28 females, with an average age of 47 ± 10 years), and 48 type 2 diabetic (25 males and 23 females, with an average age of 49 ± 12 years) patients. All specimens were from the same ethnic group with the same socioeconomic background, and all specimens were collected in accordance with relevant laws and regulations.

In the early morning of the second day, venous blood was collected from the patients and the FPG test was performed. Also, the OGTT test was performed on all subjects, and venous blood was collected after 2 h of glucose loading, and the venous blood glucose level was measured. During the OGTT test, subjects would sit and rest, and drinking coffee, tea, and other substances was prohibited. The detailed results of the blood glucose analyses are provided in Table 1.

**Table 1:** Blood glucose information includes 2 h post-load blood glucose (2 hPG) and fasting plasma glucose (FPG).

**Blood sugar target**	**Max**	**Min**	**Average**	**Standard deviation**
2 hPG(mmol/L)	11.8	5.40	9.10	1.14
FPG(mmol/L)	7.60	3.7	5.09	0.674

In this study, the diagnostic criteria for type 2 diabetes and pre-diabetes used the standards formulated in the “Guidelines for Prevention and Treatment of Type 2 Diabetes (2013 Edition)” as the reference basis (Diabetes Branch of Chinese Medical Association, 2014): (1) normal blood glucose: FPG <7.0 mmo1/L and (or) 2hPG <7.8 mmol/L; (2) diabetes: FPG > 7.0 mmol/L and (or) 2hPG > 11.1 mmol/L (patients with a diagnosis of diabetes); (3) pre-diabetes mellitus (PDM): FPG range: 6.1 ≤ FPG < 7.0 mmo1/L and (or) 7.8 mmol/L < 2hPG ≤ 11.1 mmol/L.

In this study, the sample set was divided into a training set and a prediction set at a ratio of 3:1 by using a random selection method and repeating sampling 10 times. The random division ensures that the sample sets generated every time by setting random seeds were different and can be compared with the results of multiple runs because the method remarkably influences the model robustness, and an optimal sample set was obtained. The division of the blood samples into the training set and the prediction set is presented in Table 2.

**Table 2:** Division of blood samples into the training set and prediction set.

**Sample**	**Total samples**	**Healthy group**	**Pre-diabetes group**	**Diabetes group**
Total samples	147	53	46	48
Training set	111	40	35	36
Validation set	36	13	11	12

### Collection of Near-Infrared Spectra

NIRS were acquired using a grating NIR spectrometer (XDS Rapid Content Analyzer, Foss, Denmark) with transmission accessories. The spectra acquisition range was 400–2,500 nm, and the detectors were Si (400–1,100 nm) and PbS (1,100–2,500 nm). When acquiring NIRS of the blood samples from a group of healthy, pre-diabetic, and diabetic patients, 1-mL sample portions were placed in a quartz cuvette (optical path of 1 mm), and spectra were recorded at a wavelength increment of 2 nm in the range of 400–2,500 nm. The spectral data of each sample were measured in triplicate and averaged. The laboratory temperature was 24 ± 1°C and the relative humidity was 41%.

Data preprocessing is an important factor to improve prediction accuracy (Byrne et al., 2016). Random noise is often a component of the original data, resulting in differences between the true and the measured value. To eliminate errors as much as possible, it is necessary to weaken and even eliminate various disturbance factors through various data processing methods, which lay the foundation for next data processing. Therefore, it is very necessary to preprocess the original spectra. In this study, spectral data were preprocessed by a first derivative, a second derivative, a multiple scattering correction (MSC), and a standard normal variable transform (SNV) which can be used to reduce or even remove the influence of various interference factors.

### Support Vector Machine (SVM)

SVM is a machine learning method that was developed based on dimensional theory and the statistical learning theory of Vapnik (1995). SVM is used to investigate pattern recognition and regression prediction problems with small sample sizes and can solve many practical problems, such as small sample size, nonlinearity, and high-dimensional problems. The problems of poor generalization ability and the difficult convergence of neural networks were solved by SVM. In recent years, good progress has been made in studies on disease diagnosis by using NIRS combined with SVM (Sylvain and Cecile, 2018; Afara et al., 2020).

In the SVM method, different kernel functions can generate different SVM algorithms. A radial basis function (RBF) kernel function is used to realize the modeling classification of SVM because it can process nonlinear problems. RBF is a scalar function that is symmetric along the radial direction. It is usually defined as a monotonic function of the Euclidean distance between any point x in the space and a certain center x_c_, which can be recorded as k(||x-x_c_||), and its effect is often local, that is, when x is far away from x_c_, the value of the function is very small (Sánchez, 2003). Moreover, several optimization algorithms have been adopted to optimize the internal parameters of the model, obtain better results, and increase model robustness. Kernel function optimization is mainly solved by using penalty parameter C and kernel function parameter g. Parameter optimization is implemented based on the principle of minimum mean square error. The two parameters, namely, the selected kernel function type and support vector type, determine the optimization performance of the model. No universally agreed method has been reported for the optimization of SVM parameters worldwide. At present, the common methods include test method, grid search (GS), genetic algorithm (GA), and particle swarm optimization (PSO) (Sánchez, 2003; Peng-Wei et al., 2004).

Principal component analysis was used for dimension reduction to decrease model complexity (Abdi and Williams, 2010). The data after dimension reduction determined the data of principal components with a cumulative contribution rate higher than 99%, which are used as the input of the SVM model. Moreover, the kernel function was used for SVM modeling because RBF can accurately process nonlinear problems. The penalty parameter c and kernel function parameter g were used as two important parameters of RBF. These two parameters have important control impacts on model complexity, approximation error, and measurement accuracy of the model (Schlkopf and Smola, 2001; Aljarah et al., 2018; Li et al., 2019; Yalsavar et al., 2019). The penalty parameter c in the SVM model represents the degree of the penalty of an incorrect classification under linearly inseparable situations. This parameter adjusts the preferred weights of two indexes (interval and classification accuracies) in the optimization direction. This problem is equal to the prohibition of incorrectly classified samples (overfitting) when c tends to be infinitely large. The accurate classification of samples is ignored, and the maximum interval is pursued when c tends to be 0. Relevant solutions are then not obtained, and the algorithm does not converge (underfitting). The kernel function parameter g is the first r (γ) in Equation (2), and the default value is 1/k where k is the number of categories. The value of γ is used to set the “spread” of the function when RBF is utilized as the kernel. This condition applies the data mapping distribution to the new characteristic space. The value of γ is negatively correlated with the number of support vectors which influences the training and prediction speeds.

(1) $$\begin{array}{l} K\left(x,y\right)=\exp\left\{-\gamma ||x-y|{|}^{2}\right\} \end{array}$$

### Aquaphotomics

Water, as a natural biological matrix, is composed of small molecules with a great capacity for hydrogen bonding. Water alters the absorption pattern according to the physical and chemical properties of biological systems (Tsenkova, 2008). The basis component of blood is water, and the water absorption pattern will change due to the changes in material metabolism in the human body with disease. Consequently, changes in water absorption patterns of the blood can be used to diagnose the disease. The 12 water absorption bands - WAMACS in the NIR range are presented in Table 3 (Tsenkova, 2009; Tsenkova et al., 2015, 2018; Bázár et al., 2016).

**Table 3:** Water absorption pattern in NIR range.

**WAMSCs**	**Range (nm)**	**Characteristic wavelengths (nm)**	**Assignment**	**References**
C1	1,336–1,348	1,344	υ_3_	Kondepati et al., 2008
C2	1,360–1,366		Water shell	Robertson et al., 2003
C3	1,370–1,376	1,374	υ_1_ + υ_3_	Roggo et al., 2007
C4	1,380–1,390	1,382	Water shell	Ludwig, 2001
C5	1,398–1,418	1,408, 1,416	S_0_	Donis-Gonzalez et al., 2016
C6	1,420–1,428		Water hydration	Cao et al., 2006
C7	1,434–1,444		S_1_	Cattaneo et al., 2009
C8	1,448–1,454	1,448	υ_1_ + υ_3_	Kalinin et al., 2013
C9	1,460–1,468	1,462	S_2_	Jaenicke and Lilie, 2000
C10	1,472–1,482	1,470	S_3_	Diller, 1992
C11	1,482–1,495		S_4_	Gowen et al., 2009
C12	1,506–1,516		Strongly bonded water or υ_2_	Xantheas, 1995a,b

WAMACS, water matrix coordinates.

ν_1_, symmetric stretching of first overtone of water.

ν_2_, bending of first overtone of water.

ν_3_, asymmetric stretching of first overtone of water.

*S_0−4_, (H_2_O)_1−5_*.

As water is susceptible to disturbance factors, analyzing the spectral changes of water and living systems subjected to these factors can be used to study the water matrix and other molecules in the water. The spectral ranges of water absorbance bands called water matrix coordinates (WAMAC_S_), where specific water absorbance bands related to specific water molecular conformations (water species, water molecular structures) are found with the highest probability (Tsenkova, 2009). For the first overtone of water (1,300–1,600 nm), 12 WAMACs (labeled Ci, i = 1, 12) have been experimentally discovered (each of 6–20 nm in width) and they have been confirmed by overtone calculations of already reported water bands in the infrared range (Tsenkova, 2009). The combination of the activated water bands, at which the light absorbance gets influenced by the perturbations, depicts a characteristic spectral pattern called a water spectral pattern (WASP), which reflects the condition of the whole water molecular system. It contains a huge amount of physical and chemical information for the solution because the water hydrogen bonding network is easily influenced by any kind of even subtle perturbations (Kinoshita et al., 2012) including the solutes. At the moment, even without the assignment and understanding of water absorbance bands, WASPs can be used as holistic (bio)markers for system functionality.

The trajectory of the water absorption pattern obtained under a specific disturbance can be used as a spectral pattern in the multidimensional space of the water matrix coordinates, that is, as a spectral biological indicator to distinguish substances and explain the difference in function and the structural characteristics of the two. The application of water absorption patterns in disease diagnosis can be used as a biological indicator to help us better understand the role of water in life systems, and for disease diagnosis (Mengli et al., 2015; Xiaoyu et al., 2019).

Graphically, WASP is presented as an aquagram, which is a radial graphic of the normalized absorbance of characteristic water bands. The values for the aquagram on the coordinate axis can be obtained according to Equation (1) (Tsenkova et al., 2015). Here, A_λ_ is the absorbance after multiplicative scatter correction (MSC) applied on the first derivative overtone region, μ_λ_ is the mean value of all spectra, and σ_λ_ is the standard deviation of all spectra at wavelength λ.

(2) $$\begin{array}{l} Aq_{\lambda }=\frac{A_{\lambda }-\mu_{\lambda }}{\sigma_{\lambda }} \end{array}$$

### Evaluation of Model Parameters

Accuracy, specificity, and sensitivity are used as important evaluation indexes for the SVM models. The calculation equations of different parameters are expressed as follows:

(3) $$\begin{array}{l} Accuracy=\frac{n_{correct}}{n_{total}} \end{array}$$

(4) $$\begin{array}{l} TPR=\frac{TP}{TP+FN} \end{array}$$

$$\begin{array}{l} FPR=\frac{FP}{FP+TN} \end{array}$$

In Equation (4), TPR is the sensitivity, and FPR is the specificity. *TP* represents the number of positive samples in the verification set that are accurately classified by the model, and *FN* represents the number of positive samples in the verification set that are incorrectly classified by the model. *FP* is the number of negative samples in the verification set that are incorrectly classified by the model. *TN* is the number of negative samples in the verification set that are accurately classified by the model.

## Result and Discussion

### Spectral Analysis

**Figure 1:**
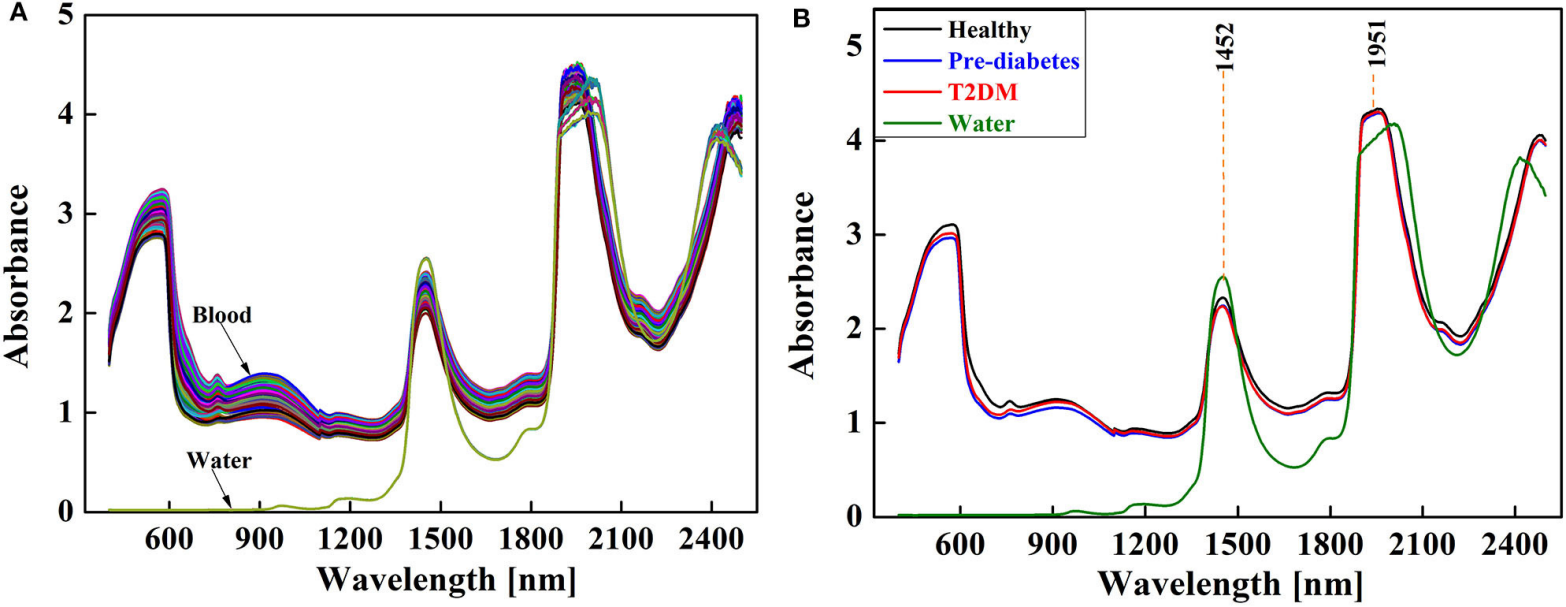
NIR spectra of the blood samples: **(A)** raw and **(B)** average spectra.

NIRS of the blood and water samples are shown in Figure 1 and the raw spectra of the blood samples are shown in Figure 1A. In the figure, there are two main absorption peaks at 1,452 and 1,951 nm in the NIR region, which are in accordance with the fingerprint region of water as reported in the literature. The absorption peak at 1,452 nm is the first overtone of the O-H stretching vibration in water, and that at 1,951 nm is the combination of O-H stretching and bending vibrations in water (Sakudo, 2016; Bishop and Neary, 2018; Pasquini, 2018).

In addition to water as the main component of blood, cholesterol, triglycerides, glucose, proteins, and other organic compounds are found in blood, but the absorption region of these substances are masked by the absorption of water molecules. The shape and trend of the NIRS of the healthy group, pre-diabetes group, and diabetes group are very similar, but the absorption intensity is dissimilar (shown in Figure 1B). It can be observed that the absorption intensity is healthy group > diabetes group > pre-diabetes group in the 400–1,200 nm region, and at 1,452 nm it changes to healthy group > pre-diabetes group > diabetes group, which is probably due to the changes in water and other organic compounds caused by changed blood glucose concentration.

### Result of SVM Model

The results of the SVM model based on different preprocessing methods are shown in Table 4. It was concluded that the first derivative had the best preprocessing effect, so the first derivative was chosen as the preprocessing method for this data. GS, GA, and PSO were used to optimize c and g in this study. The optimum c and g values were selected based on the principle of highest accuracy, which is gained by cross-verification of the leave-one-out method. It follows from Table 5 that the model has the best effect under the condition of GA (c = 11.62, g = 0.009346) optimization. The accuracy, specificity, and sensitivity are: 97.22%, 95.65% (22/23), 100% (13/13), respectively with the prediction set.

**Table 4:** Results of SVM model based on different preprocessing methods.

**Pre-treatment**	**C**	**g**	**CV accuracy (%)**	**Accuracy (%)**	**Sensitivity**	**Specificity**
Untreated	147.0	0.0068	77.04	91.66	95.65 (22/23)	84.62 (11/13)
**First derivative**	**16.00**	**0.006801**	**90.99**	**97.22**	**95.65 (22/23)**	**100.0 (13/13)**
Second derivative	48.50	0.003963	90.99	94.44	95.65 (22/23)	92.00 (12/13)
Msc	48.50	0.003906	80.18	94.44	95.65 (22/23)	92.00 (12/13)
Snv	27.85	0.006801	79.27	94.44	95.65 (22/23)	92.00(12/13)

*The bold font represents the best results*.

**Table 5:** Results of SVM model based on different optimization algorithms.

**Optimization algorithms**	**C**	**g**	**CV accuracy (%)**	**Accuracy (%)**	**Sensitivity**	**Specificity**
GS	16.00	0.006801	90.99	97.22	95.56 (22/23)	100.0 (13/13)
**GA**	**11.62**	**0.009346**	**92.80**	**97.22**	**95.65 (22/23)**	**100.0 (13/13)**
PSO	1.500	1.700	9.580	91.66	91.31 (21/23)	92.00(12/13)

*The bold font represents the best results*.

**Figure 2:**
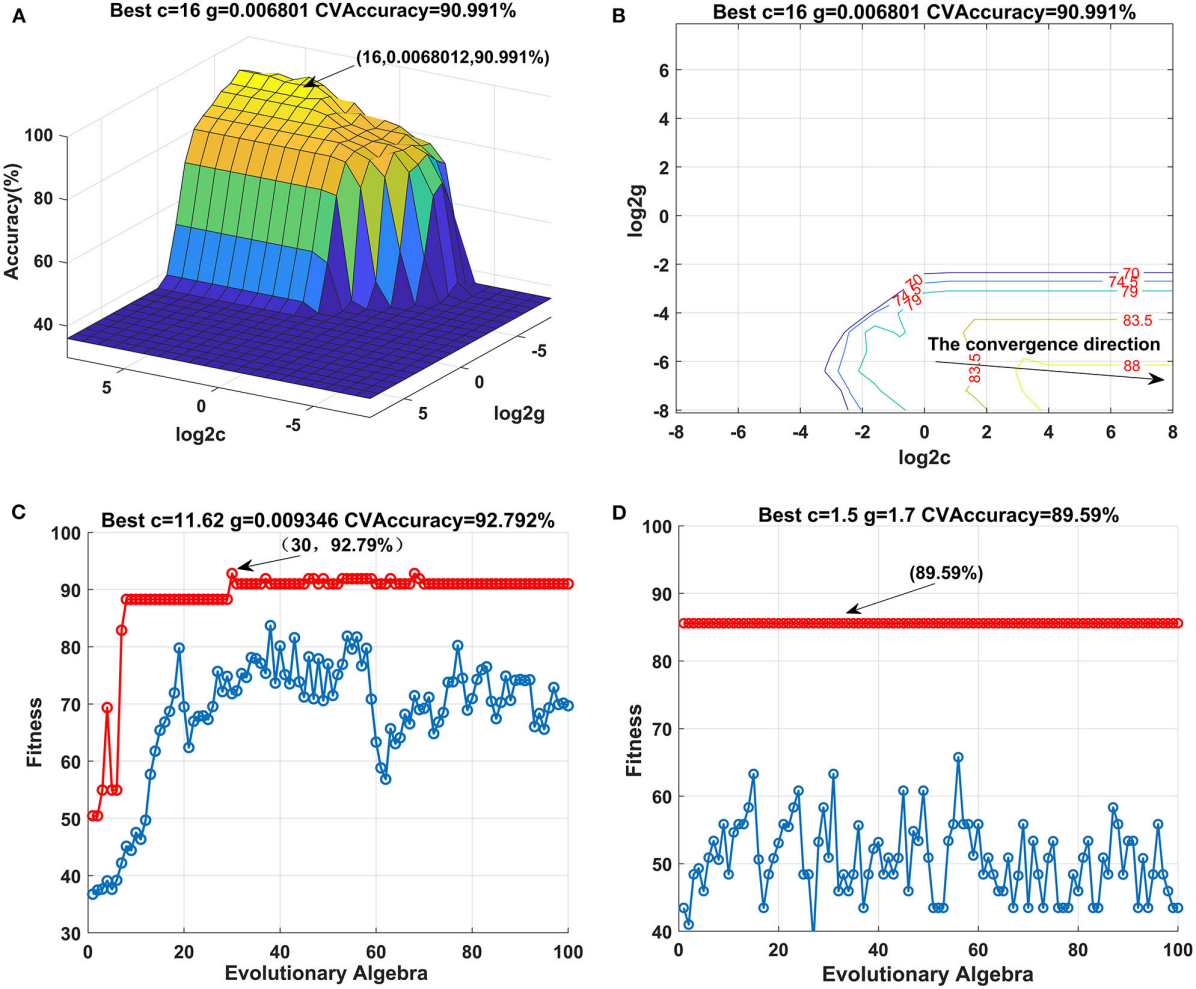
Optimization result map for healthy, pre-diabetic, and type 2 diabetic samples: **(A,B)** optimization result map for GS; **(C)** optimization result map for GA; and **(D)** optimization result map for PSO.

The optimal results of the healthy, pre-diabetic, and type 2 diabetic patient samples are shown in Figure 2. The 3D diagram of the optimization results for c and g using the GS methods is shown in Figure 2A. The contour map in Figure 2A projected onto a 2D plane is shown in Figure 2B. The contour map shows that c and accuracy rate gradually increase, and the gradient gradually converges from left to right. The highest accuracy rate of interactive verification is 90.99% when the penalty parameter c = 16 and the kernel function parameter g = 0.006801. The optimization results using GA are shown in Figure 2C. The accuracy rate continuously increases when the population evolution algebra increases from 0 to 30, and the population reaches its saturation point at 30. Therefore, c = 11.62 and g = 0.009346 are the optimal results with the highest accuracy rate of the interactive verification of 92.80% when the population evolution algebra is 30. The optimization results for PSO are shown in Figure 2D. Results show that the accuracy rate is saturated at all times when the population evolution algebra is between 0 and 100. Therefore, c = 1.5 and g = 1.7 are the optimal results with the highest accuracy rate of the interactive verification of 89.58% when the population evolution algebra is between 0 and 100.

**Figure 3:**
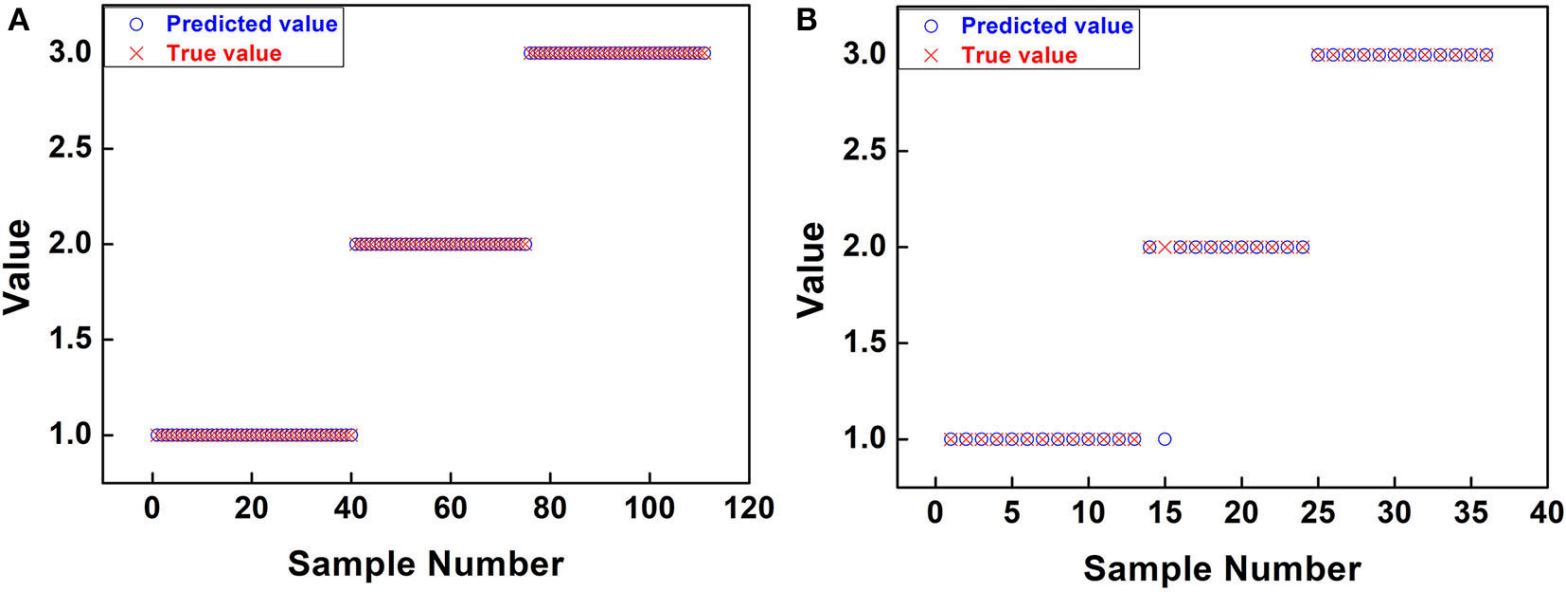
Graphs for the estimated class values (y axis) vs. the number of samples (x axis). **(A)** Training set. **(B)** Prediction set.

Graphs for the estimated class values (y axis) vs. the number of samples (x axis) are shown in Figure 3. The best fit between the true and predicted values of the training set is shown in Figure 3A. The fitting effect of the true value and predicted value of the prediction set is shown in Figure 3B, and only one outlier sample is found.

### Result of Aquaphotomics

**Figure 4:**
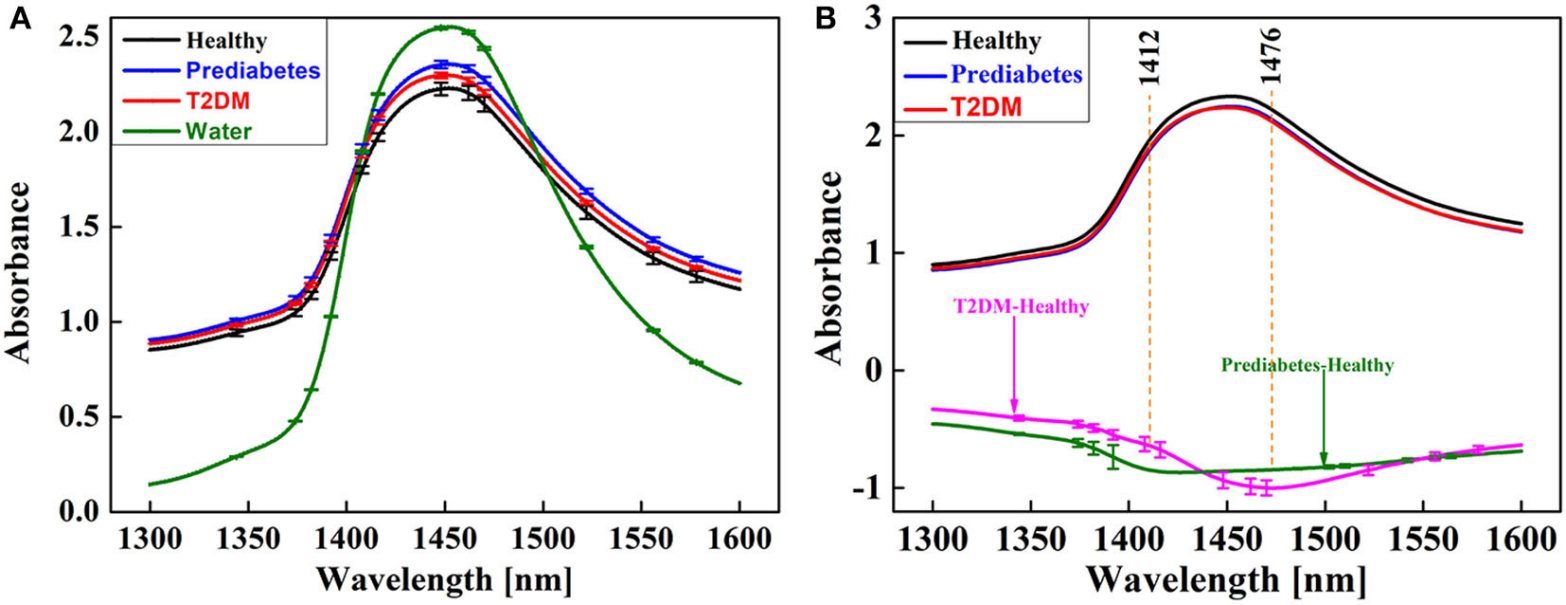
Average spectra of the blood samples in the 1,300–1,600 nm region: **(A)** raw spectra; **(B)** difference spectra of pre-diabetes and type 2 diabetes.

The average and corresponding different spectra of healthy, pre-diabetic, type 2 diabetic, and pure water in 1,300–1,600 nm are shown in Figure 4. It shows that the spectra of healthy, pre-diabetic, and type 2 diabetic almost overlapped, except at 1,450 nm where large differences were observed. The resulting spectra obtained by subtracting the healthy spectra from the pre-diabetic and type 2 diabetic spectra showed a maximum negative peak at 1,412 and 1,476 nm, which are attributable to the stretching vibration peak of water molecules without hydrogen bonds (1,412 nm) and water molecules with three hydrogen bonds (1,476 nm) (Tsenkova, 2009; Tsenkova et al., 2018; Xueguang et al., 2018). Error bars represent the fluctuation range of absorbance of different individuals. Due to the influence of noise and individual variation, it was difficult to diagnose diabetes in the early stages when the differences from the raw spectra were barely visible, but in the range of individual differences, the difference between pre-diabetes and type 2 diabetes can be observed from the differential spectra.

**Figure 6A** shows the results of the second derivative of the raw spectra and the corresponding different spectra of the healthy, pre-diabetic, and type 2 diabetic samples. The second derivative spectra can effectively expand the resolution of the spectra and find the differences among the spectra of healthy, pre-diabetic, and type 2 diabetic. More distinctive peaks appeared after the second derivative transformation, including a maximum positive peak centered at 1,382 nm and a maximum negative peak centered at 1,416 nm. The peak at 1,382 nm was ascribed to the solvent layer of water and 1,416 nm was ascribed to water molecules without hydrogen bonds (Tsenkova, 2009; Tsenkova et al., 2018; Xueguang et al., 2018). The resulting spectra obtained by subtracting the healthy spectra from the pre-diabetic and type 2 diabetic spectra showed the differences at 1,408 nm (water molecules that do not contain hydrogen bonds), 1,416 nm (water molecules without hydrogen bonds), 1,448 nm (the solvated layer of water), 1,462 nm (water species containing two hydrogen bonds), 1,470 nm (unknown) (Tsenkova, 2009; Tsenkova et al., 2018; Xueguang et al., 2018). Error bars represent the fluctuation range of absorbance of different individuals. In the range of individual differences, the difference between pre-diabetes and type 2 diabetes can be observed from the corresponding different spectra of the second derivatives.

**Figure 5:**
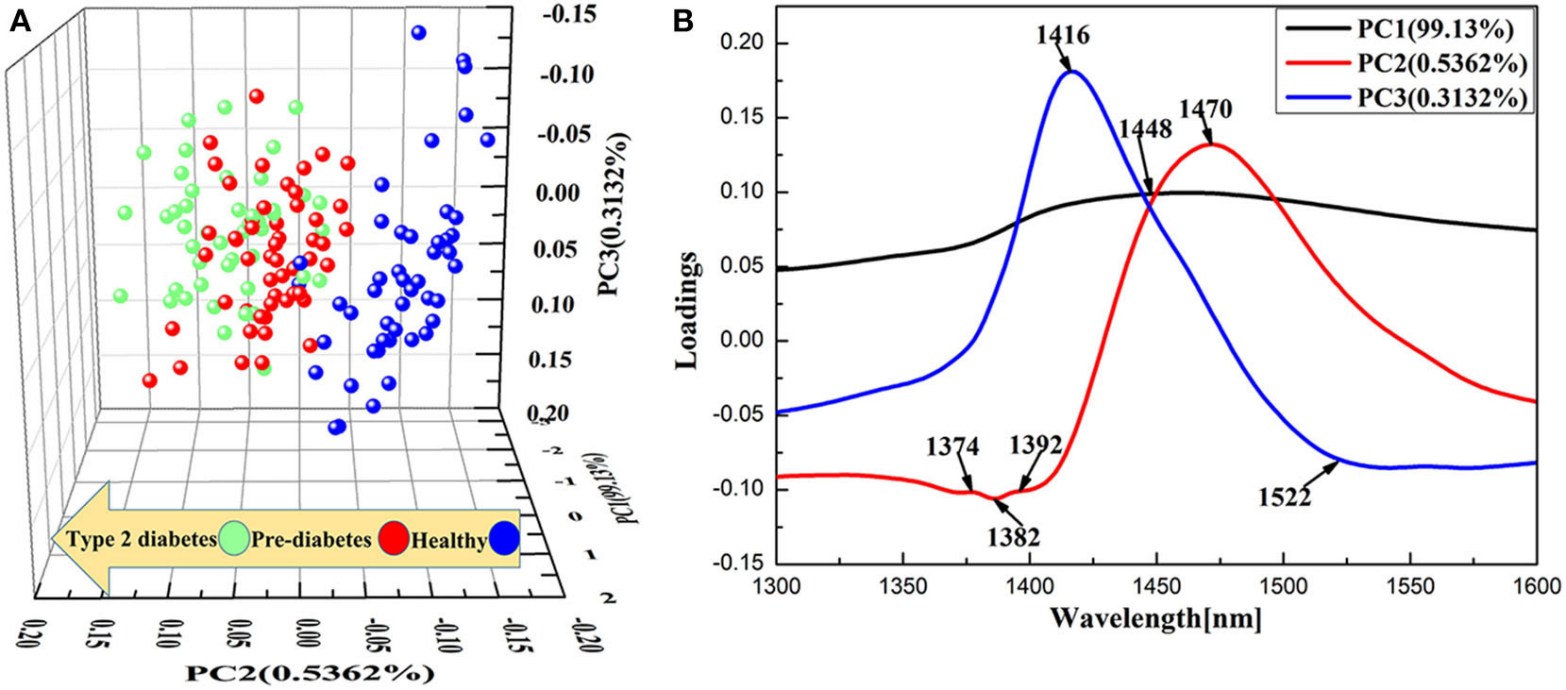
PCA results of NIR spectra (1,300–1,600 nm) collected from all samples. **(A)** PCA 3D score plot. **(B)** PCA loading plot.

In general, NIRS are highly correlated and cause data redundancy to a certain extent. Principal component analysis (PCA) was applied to the NIRS of all samples from 1,300 to 1,600 nm because of its ability for data reduction. Figure 5A presents the three-dimensional score plots, which show the projection of raw data onto the 3D plane of the first three principal components of PCA. The cumulative explained variance of the first three principal components was 99.98%, indicating that the first three principal components were able to reflect most of the essential characteristics of the raw data. As shown in Figure 5A, from healthy, pre-diabetes, and type 2 diabetes, there was a trend along the PC2-coordinate from negative to positive values, suggesting that as diabetes progresses the original water structures of the whole blood were gradually disrupted. The water structures of the pre-diabetes gradually approached the water structures of the type 2 diabetes as diabetes progressed. Figure 5B is the loading plot of the first three principal components of PCA. PC3 showed higher loading values at 1,416 nm, whereas the loading values of PC1 and PC2 mainly were highest at 1,374, 1,382, 1,392, 1,448, 1,470, and 1,522 nm. In addition to the aforementioned distinctive peaks, the peak at 1,416 nm represented water molecules without hydrogen bonds and 1,522 nm was strongly bound water, water species with four hydrogen bonds absorbed in 1,482–1,495 nm (Esquerre et al., 2009; Gowen et al., 2009; Tsenkova, 2009).

Several studies have shown that there are 12 characteristic bands in the first overtone region of water (1,300–1,600 nm). As shown in Table 3, there were eight peaks, including 1,344, 1,374, 1,382, 1,408, 1,406, 1,448, 1,462, and 1,470 nm, in the NIRS of healthy, pre-diabetes, and type 2 diabetes samples found within these 12 characteristic bands. Each band corresponded to a peak, except for the two peaks in the C5 region. The 12 characteristic wavelengths were selected (1,344, 1,374, 1,382, 1,392, 1,408, 1,416, 1,448, 1,462, 1,470, 1,522, 1,556, 1,578 nm) according to the results of spectral variance analysis. PCA analysis to construct aquagrams for the evaluation of water structural changes in whole blood shows how diabetes progresses. The selected 12 characteristic wavelengths were the WAMACs of the entire complex system of the whole blood in healthy, pre-diabetes, and type 2 diabetes patients, and the changes in absorbance of these 12 characteristic wavelengths corresponded to the WASP of the samples.

**Figure 6:**
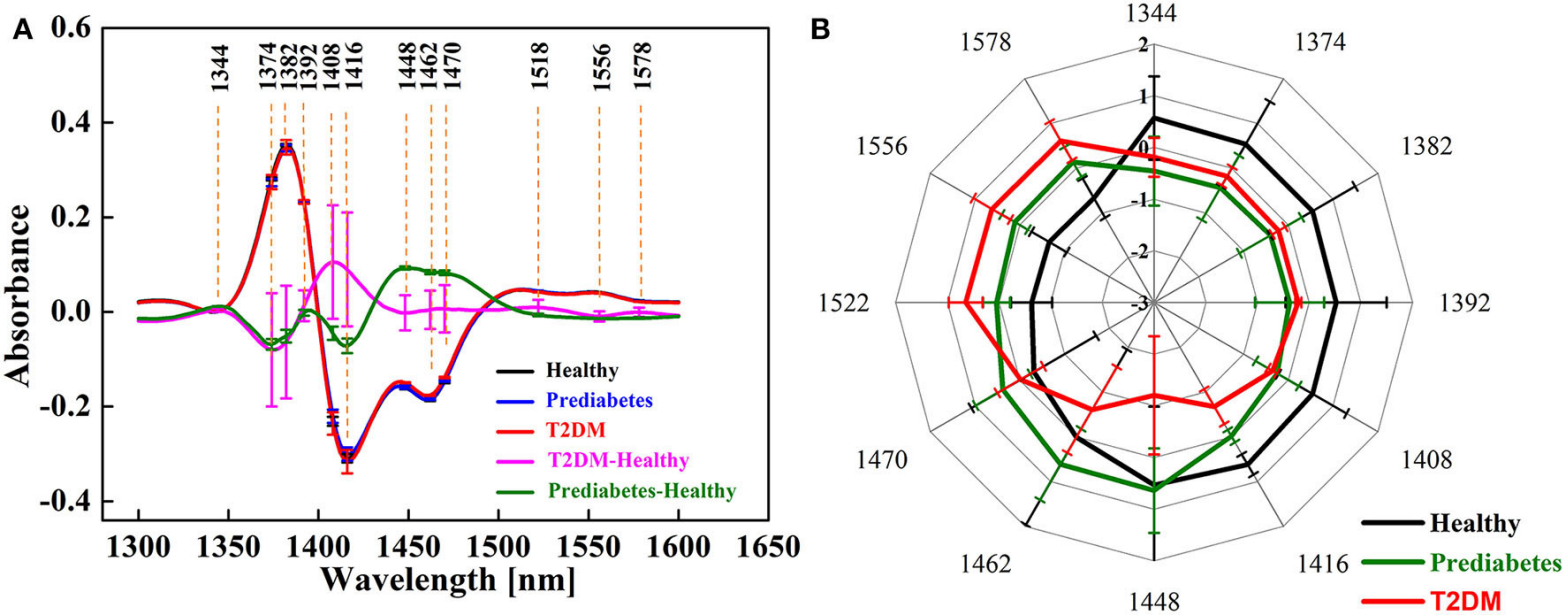
Second derivative spectra and aquagram: **(A)** average and difference spectra in the 1,300–1,600 nm region; **(B)** aquagram of healthy, pre-diabetes, and type 2 diabetes samples in 12 fingerprint regions of water.

The radar chart called an “aquagram” was drawn according to Equation (2) above as shown in Figure 6B. Error bars represent the fluctuation range of absorbance of different individuals. Within the error fluctuation range as shown in Figure 6B, water absorption patterns for the early diagnosis of diabetes are feasible and the differences were observed in aquagrams. The aquagrams of the healthy, pre-diabetes, and diabetes groups are clearly biased differently. The healthy group has the strongest absorbance at six WAMACS of 1,344 nm (anti-symmetric stretching fundamental frequency vibration), 1,374 nm (symmetrical and anti-symmetrically stretching fundamental frequency vibration), 1,382 nm (solvent layer of water), 1,392 nm (trapped water), 1,408 nm (water molecules that do not contain hydrogen bonds), and 1,416 nm (water molecules without hydrogen bonds).The absorbance of blood in the diabetes group was evidently closer to the center of the aquagram, and that in the pre-diabetes group was far away from the center of the aquagram in the 1,448 nm region (the solvated layer of water). In addition, the average intensity of absorbance of the pre-diabetic group at 1,462 nm (water species containing two hydrogen bonds) and 1,470 nm (unknown) was stronger than the diabetes group, while the absorbance of blood in the diabetes group at 1,522 nm (water species containing four hydrogen bonds), 1,556 nm (unknown) and 1,578 nm region (unknown) was stronger than that in the pre-diabetes group.

The 1,462 and 1,470 nm bands are found in sugar-water systems, and are also similar to the bands we found in the region above 1,500 nm (Esquerre et al., 2009; Gowen et al., 2009; Tsenkova, 2009). Also, the result shows that there is a trend in the increased concentration in the aquagrams of water-sugar solutions which is consistent with the findings of Bázár et al. (2015). The error bars on the aquagram show a large overlap between different classes, but there is no overlap in the 1,416 nm band. Therefore, judging the progress of diabetes by the number of hydrogen bonds is affected by individual differences to a certain extent, except at 1,416 nm (water molecules without hydrogen bonds) where large differences were observed without overlap.

It can be observed that the symmetric and antisymmetric stretching fundamental frequency vibrations of the water molecules are much stronger in the healthy group. Water molecules without hydrogen bonds indicated that the Van der Waals force played a significant role in the water molecules of blood, which showed that healthy people had normal blood glucose metabolism. In this case, the H in the hydrogen bonds of glucose that participates in the formation of hydrogen bonds is almost negligible, and only a small amount of H in hydrogen bonds of water molecule participates in hydrogen bonding. Therefore, the absorbance of blood in the healthy group at 1,408 nm (water molecules without hydrogen bonds) and 1,416 nm (water molecules without hydrogen bonds) was higher than that in the pre-diabetes group and diabetes group.

**Figure 7:**
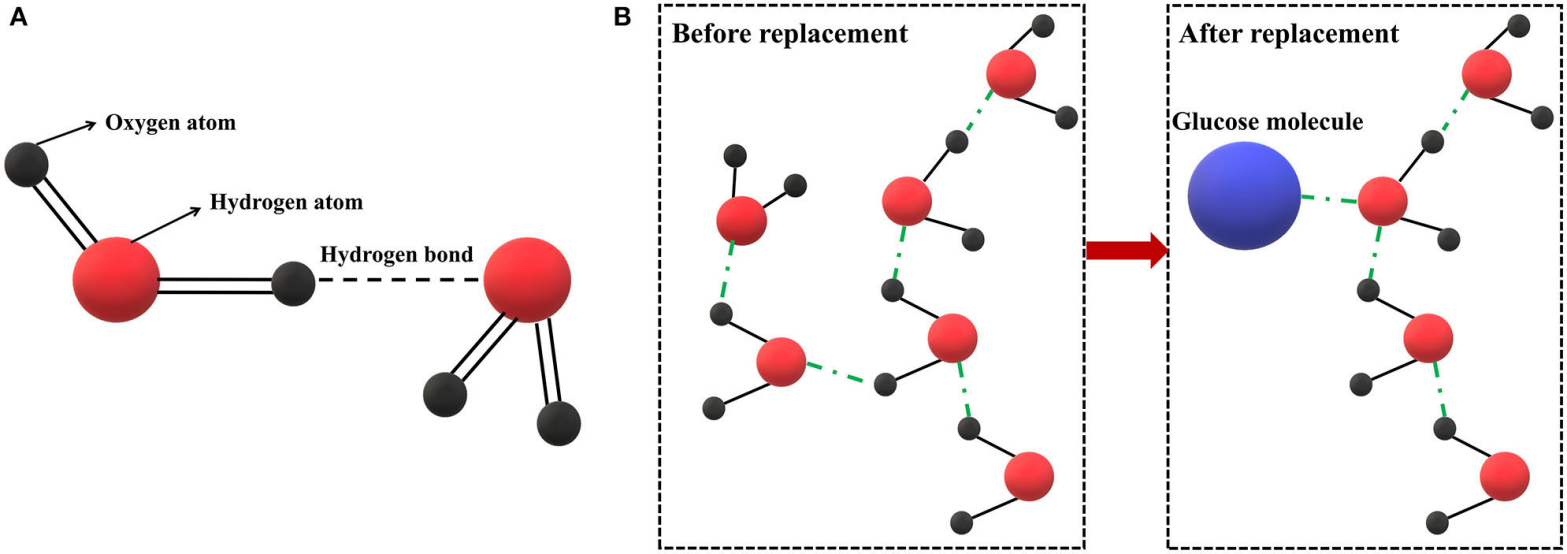
Hydrogen bond interaction: **(A)** schematic diagram of hydrogen bonding between water molecules; **(B)** replacement effect of glucose on water in blood glucose.

The number of hydrogen bonds contained in the water species are in the order of diabetes group > pre-diabetes group > healthy group, indicating that as the blood glucose metabolic dysfunction becomes more serious, the H in blood glucose replaces the H in water to participate in the formation of hydrogen bonds (shown in Figure 7). This indicates that the hydrogen of -OH in glucose competes with the hydrogen of -OH in water. The water environment in human blood has been changed by the aggravation of abnormal blood glucose metabolism. The H of -OH in glucose replaces the H of -OH in water to participate in hydrogen bonding and forms many glycosylation products such as glycated hemoglobin and glycated albumin (Yun, 2009). The interaction between high concentrations of blood sugar and other blood components in the long-term leads to serious effects on the health of the human body owing to the further aggravation of the disorder of glucose metabolism. Therefore, the number of non-bonded water molecules can be used as a biomarker for the early diagnosis of diabetes.

## Discussion

According to the results of the SVM model, the accuracy of the early diagnosis of diabetes can reach 97%. However, the model is not interpretable and cannot explain the process of diabetes occurrence and development, nor can confirm whether blood composition has changed, resulting in the corresponding changes of spectral characteristics. However, the difference in water absorption patterns of blood among the healthy group, pre-diabetes group, and diabetes group can be visually observed using the aquaphotomics method. With the intensification of blood glucose metabolism disorders, the water environment in the blood changes significantly. The H of -OH in glucose slowly replaces the H of -OH in water to participate in hydrogen bonding. The progress of diabetes can be observed in water absorption patterns at 1,408, 1,416, 1,462, and 1,522 nm which are assigned to water molecules with a different number of hydrogen bonds. The displacement effect of glucose on water has been discovered and experimentally verified in aqueous glucose solution (Brady and Schmidt, 1993; Yun, 2009; Cong et al., 2012; Xiaoyu et al., 2017; Sae et al., 2018; Arai and Shikata, 2019; Beganović et al., 2020). Therefore, it is speculated that this phenomenon is also applicable to complex solution systems such as blood, and this experiment has observed the displacement effect of glucose on the water in blood, and is applied to the early diagnosis of diabetes.

Pre-diabetes is a condition defined as having blood glucose levels above normal but below the defined threshold of diabetes. It is considered to be an at-risk state, with a high chance of developing diabetes (Tabák et al., 2012). While pre-diabetes is commonly an asymptomatic condition, it is always present before the onset of diabetes. The elevation of blood sugar is a continuum and hence pre-diabetes cannot be considered an entirely benign condition. Therefore, pre-diabetes is a necessary stage for diabetic patients. The early diagnosis of diabetes is an important way to reduce morbidity, complications, and mortality, and has important significance for the clinical evaluation and prevention of diabetes (Cefalu et al., 2014; Yi et al., 2014; Huang et al., 2016; Khokhar et al., 2017).

This study, different to those conducted previously, has used near-infrared spectroscopy combined with machine learning and aquaphotomics for the early diagnosis of diabetes. The diagnosis accuracy has reached 97%. Differences in water absorption patterns were analyzed, and the specific features of the water spectra that can be used as a biomarker for the early diagnosis of diabetes were found. Besides, the occurrence and development of diabetes were explained at the molecular level. Specifically, as the disorder of blood glucose metabolism intensifies, the water environment of blood changes significantly. The H of -OH in glucose replaces the H of -OH in water to participate in hydrogen bonding, and the severity of diabetes can be reflected via the number of hydrogen bonds contained in the water species.

## Conclusion

In this study, the near-infrared spectra of blood samples from healthy, pre-diabetes, and diabetes groups were collected and it was found that the raw near-infrared spectra were not significantly different. However, after the second-order derivative was used to improve the spectral resolution, significant differences were found in the 1,400–1,500 nm region, which shows that water absorption patterns could be used for the early diagnosis of diabetes. Therefore, NIRS combined with machine learning and aquaphotomics were used for the early diagnosis of diabetes in this paper. The results show that the optimization of different preprocessing methods and optimization algorithms (GS, GA, PSO) can greatly improve the accuracy rate of the SVM model, and a high accuracy rate of 97% was obtained by the SVM model for recognizing the healthy, pre-diabetes, and diabetes groups. Difference of water absorption patterns in blood was analyzed by aquaphotomics method, and results show that the number of hydrogen bonds contained in the water species decreased in the order of diabetes group > pre-diabetes group > healthy group, which indicated a significant change in the water environment between the groups. Owing to the dysfunction of the blood glucose metabolism, the H of -OH in glucose replaces the H of -OH in water to participate in hydrogen bonding, and the severity of the diabetes can be reflected via the number of hydrogen bonds.

## Data Availability Statement

The raw data supporting the conclusions of this article will be made available by the authors, without undue reservation.

## Ethics Statement

The studies involving human participants were reviewed and approved by Ethics Committee of the First Affiliated Hospital of Jinan University. The patients/participants provided their written informed consent to participate in this study.

## Author Contributions

FH and YL: conceptualization of the project. YL and LG: data curation, data analysis and validation, and writing the original draft. YL, LG, and LL: laboratory investigation. FH and ZC: project administration and funding resources. YL, LG, CY, PG, and FH: writing—review and editing. All authors contributed to the article and approved the submitted version.

## Conflict of Interest

LG was employed by the company Guangdong Hongke Agricultural Machinery Research & Development Co., Ltd. The remaining authors declare that the research was conducted in the absence of any commercial or financial relationships that could be construed as a potential conflict of interest.
